# Transcriptome dynamics and molecular cross-talk between bovine oocyte and its companion cumulus cells

**DOI:** 10.1186/1471-2164-12-57

**Published:** 2011-01-24

**Authors:** A Regassa, F Rings, M Hoelker, U Cinar, E Tholen, C Looft, K Schellander, D Tesfaye

**Affiliations:** 1 Institute of Animal Science, Animal Breeding and Husbandry Group, University of Bonn, Germany

## Abstract

**Background:**

The bi-directional communication between the oocyte and its companion cumulus cells (CCs) is crucial for development and functions of both cell types. Transcripts that are exclusively expressed either in oocytes or CCs and molecular mechanisms affected due to removal of the communication axis between the two cell types is not investigated at a larger scale. The main objectives of this study were: 1. To identify transcripts exclusively expressed either in oocyte or CCs and 2. To identify those which are differentially expressed when the oocyte is cultured with or without its companion CCs and vice versa.

**Results:**

We analyzed transcriptome profile of different oocyte and CC samples using Affymetrix GeneChip Bovine Genome array containing 23000 transcripts. Out of 13162 genes detected in germinal vesicle (GV) oocytes and their companion CCs, 1516 and 2727 are exclusively expressed in oocytes and CCs, respectively, while 8919 are expressed in both. Similarly, of 13602 genes detected in metaphase II (MII) oocytes and CCs, 1423 and 3100 are exclusively expressed in oocytes and CCs, respectively, while 9079 are expressed in both. A total of 265 transcripts are differentially expressed between oocytes cultured with (OO + CCs) and without (OO - CCs) CCs, of which 217 and 48 are over expressed in the former and the later groups, respectively. Similarly, 566 transcripts are differentially expressed when CCs mature with (CCs + OO) or without (CCs - OO) their enclosed oocytes. Of these, 320 and 246 are over expressed in CCs + OO and CCs - OO, respectively.

While oocyte specific transcripts include those involved in transcription (*IRF6, POU5F1, MYF5, MED18*), translation (*EIF2AK1, EIF4ENIF1*) and CCs specific ones include those involved in carbohydrate metabolism (*HYAL1, PFKL, PYGL, MPI*), protein metabolic processes (*IHH, APOA1, PLOD1*), steroid biosynthetic process (*APOA1, CYP11A1, HSD3B1, HSD3B7*). Similarly, while transcripts over expressed in OO + CCs are involved in carbohydrate metabolism (*ACO1, 2*), molecular transport (*GAPDH, GFPT1*) and nucleic acid metabolism (*CBS, NOS2*), those over expressed in CCs + OO are involved in cellular growth and proliferation (*FOS, GADD45A*), cell cycle (*HAS2, VEGFA*), cellular development (*AMD1, AURKA, DPP4*) and gene expression (*FOSB, TGFB2*).

**Conclusion:**

In conclusion, this study has generated large scale gene expression data from different oocyte and CCs samples that would provide insights into gene functions and interactions within and across different pathways that are involved in the maturation of bovine oocytes. Moreover, the presence or absence of oocyte and CC factors during bovine oocyte maturation can have a profound effect on transcript abundance of each cell types, thereby showing the prevailing molecular cross-talk between oocytes and their corresponding CCs.

## Background

The bi-directional communications between the oocyte and its companion cumulus cells (CCs) is crucial for the development and functions of both cell types [1-3]. This dialogue is vital for the oocyte to acquire meiotic and developmental competence and for proliferation and differentiation of CCs [1,3-9]. The oocyte regulates proliferation [2,10-13], apoptosis [14], luteinization [13,15], metabolism [16] and expansion [17,18] of CCs through oocyte secreted factors (OSFs) such as growth and differentiation factor 9 (*GDF9*), bone morphogenetic protein 15 (*BMP15*) and possibly others. Competent oocytes also influence the expression of cumulus specific biochemical markers that might be crucial for cumulus expansion and thereby achieve maturation and successful development [17-19]. CCs play an important role in the utilization of energy substrates by oocyte [20], prevent the oocyte from oxidative stress induced apoptosis [21,22] and stimulate glutathione synthesis [23,24] during in vitro maturation. The ability of the oocyte to form male pronuclei after fertilization strongly depends on the presence of CCs during maturation [25-27] and fertilization [28-30]. Fertilization and development to a healthy blastocyst is limited by the oocyte quality [31] and CCs play a critical role in determining oocyte developmental potential both before and after ovulation [32].

The interaction between cumulus-granulosa cell derived factors such as kit ligand and oocyte secreted *GDF9 *is essential for oocyte growth [33,34]. This dialogue between the oocyte and CCs is accomplished mainly through the gap junction type of intercellular communication [35] and the presence of this junction supports oocyte competence in vitro [36]. For instance, complete removal of CCs before in vitro maturation or blockage of gap junction inhibits oocyte maturation [37]. Similarly, inhibition of these functional coupling using gap junction inhibitors significantly reduces developmental competence [38].

Developmentally competent oocytes are selected based on the number and compactness of the surrounding CCs layers [39,40] as oocytes that fail to expand their CCs can't ovulate and/or are infertile [41-44]. There is notable species level difference regarding the source and identity of cumulus expansion enabling factor (CEEF). While OSFs, like *GDF9 *and *BMP15 *are believed to be CEEF in rat [45], their presence is not mandatory for bovine [46] and porcine [47] CCs expansion in vitro as oocytectomised complexes (CC - OO) expands as equally as the intact ones.

High expression of hyaluronan synthase 2 (*HAS2*), inhibin beta A (*INHβA*), epidermal growth factor receptor (*EGFR*), geremlin (*GREM1*), beta cellulin (*BTC*), cell cycle division 44 (*CD44*), tumor necrosis alpha induced protein 6 (*TNFAIP6*) and prostaglandin synthase 2 (*PTGS2*) have been associated with developmental competence of oocytes and suggested as predictors of embryo quality in women [40] and cow [48]. On the contrary, higher expressions of cysteine proteinases *cathepsin B*, *S*, *K *and *Z *have been associated with incompetent bovine oocytes [49].

Although studies were conducted to identify molecular biomarkers for developmentally competent bovine oocytes, large scale expression data on oocyte or CCs specific transcripts is still lacking. Removal of oocyte-CCs communication axis during in vitro maturation reduces CCs expansion and thereby affects oocyte developmental competence but the effect of removing this communication axis on their gene expression is poorly understood. Furthermore, genes differentially expressed between germinal vesicle (GV) and metaphase II (MII) stage CCs are not identified and functional changes associated with those differentially expressed genes are not characterized. Identification of transcripts that are exclusively and commonly expressed between the oocyte and its companion CCs and those that are affected when the oocyte and its companion CCs mature in the presence or absence of one or the other would enhance our understanding of the molecules and biological processes that are involved in oocyte-CCs dialogue and oocyte maturation.

Therefore, this study was conducted to 1. Identify transcripts that are co and exclusively expressed between the oocyte and CCs, 2. Enumerate those which are significantly affected when the two cell types mature with or without the other and 3. Identify significantly changed biological processes during the transition of CCs from GV to MII stage.

## Methods

### Sample collection

Bovine ovaries were collected from local abattoirs and transported to the laboratory within 2-3 hours (hrs) in a thermo flask containing 0.9% physiological saline solution at 39°C. Before aspiration of COCs, the ovaries were washed twice in 70% ethanol. COCs were aspirated from antral follicles having 2-8 mm diameter using 5 ml syringe attached to 18 gauge needle. The aspirated follicular fluid was collected in 50 ml sterilized tube at 35°C and allowed to precipitate for 15 min. COCs with evenly granulated cytoplasm surrounded by multiple layers of CCs were picked using glass-pipette and washed three times in drops of modified parker medium (MPM) supplemented with 12% estrus cow serum (OCS).

**Figure 1:**
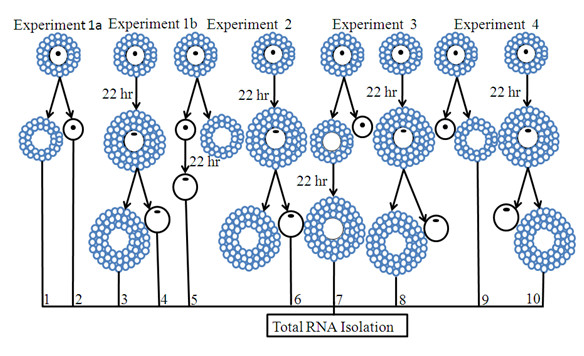
**Diagrammatic illustration of the experimental design showing groups that were used for total RNA isolation, cDNA synthesis, array hybridization and quantitative and semi-quantitative RT-PCR validation of the array data**. Numbers, 1 - 10, under each oocyte and CCs figure, represent the samples that were used for RNA isolation, cDNA synthesis and array hybridisation, as explained in materials and methods section. For all experimental groups, the samples were derived from GV stage COCs.

**Figure 2:**
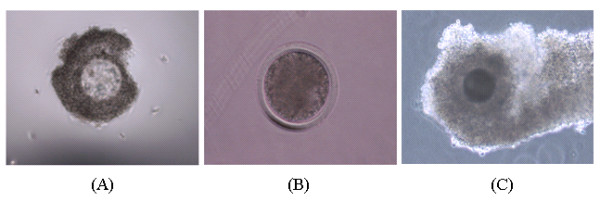
**Representative images showing CCs from which the enclosed oocyte was completely removed at GV stage using oocytectomy (A) and the oocyte from which the companion CCs were completely removed mechanically at GV stage using pipettes (B) and intact COC (C)**. Images were taken using Leica DM-IRB microscope with a magnification of 100×.

In order to increase the homogeneity of the experimental samples, COCs were further screened for developmental competence using brilliant cresyl blue (BCB) staining as described in [50-52]. BCB positive (BCB^+^) COCs were assigned randomly into the following four experiments. Each experiment had three pools of biological replicates; each replicate containing 50 oocytes or CCs samples (Figure 1). In experiment 1a, CCs were mechanically removed from oocytes at GV stage by repeated in and out pipetting and the resulting denuded oocytes (DOs), designated by number 1 and their companion CCs by number 2, were frozen. In experiment 1b, intact COCs were cultured; their companion CCs were mechanically removed and the resulting denuded MII oocytes, designated by number 4 and their companion CCs by number 3, were frozen. In experiment 2, CCs were mechanically removed from their enclosed oocytes at GV stage and the resulting (OO - CCs), designated by number 5 and other intact COCs (OO + CCs) were cultured separately. After 22 hr culture period, CCs were mechanically removed from the intact oocytes and the resulting denuded MII oocytes (number 6) were frozen. In experiment 3, oocytes were micro surgically removed from their companion CCs at GV stage as described previously [17,46,47]. Briefly, using micromanipulators with a holding pipette (20 pm diameter hole) and a fine glass needle, the complex is held under negative pressure and the oocyte pierced through both sides of the zona pellucida. The contents of the oocyte are then aspirated through the holding pipette while removing the glass needle, resulting in temporary deformation of the zona pellucida. Almost all of the oocyte contents are removed in this way, leaving an intact complex without the oocyte, the oocytectomised complex (CCs - OO). The resulting complexes (CCs - OO), designated by number 7 and other intact complexes (CCs + OO) were cultured. After 22 hr culture, oocytes were micro surgically removed from their intact cumulus oocyte complexes (CCs + OO), as described above and the resulting MII CCs without their enclosed oocytes, designated by number 8 were frozen. In experiment 4, CCs were mechanically removed from their enclosed oocytes both at GV (number 9) and MII stages (number 10) and frozen for subsequent total RNA isolation. In both cases, cells were cultured for 22 hrs and each experiment was repeated three times. Complete removal of either cell from one or the other was confirmed by microscopic examination of the corresponding samples (Figures 2A and 2B) as described in [53]. Cells were cultured in groups of 50 in 400 μl MPM medium supplemented with 12% estrus cow serum and 10 μg mL^-1 ^FSH for 22 hr at 39°C in an incubator with humidified atmosphere containing 5% CO_2_. Samples were stored at -80°C until subsequent RNA isolation.

### RNA isolation and array processing

In all experiments, always triplicate pools of oocytes or their corresponding CCs (each pool contains 50 oocytes or their corresponding CCs), were used for RNA isolation and subsequent downstream analysis. The total oocytes or CCs samples used for this study correspond to 150 oocytes or their respective CCs per each experiment.

Total RNA was isolated using PicoPure RNA Isolation Kit according to the manufacturer's instruction (Arcturus Bioscience Mt. View CA). Briefly, the samples were extracted using 100 μL of extraction buffer and resuspended by pipetting gently. They were incubated for 30 min at 42°C and centrifuged at 3,000 × *g *for two min. One hundred μL of 70% ethanol was pipetted to the cell extract and mixed well by pipetting up and down. Two hundred μL conditioning buffer was pipetted onto the purification column filter membrane and the columns were incubated with conditioning buffer for 5 min at room temperature. The purification columns were centrifuged in the provided collection tube at 16,000 × *g *for 1 min and 50 μL of 70% ethanol was pipetted into the cell extract. The cell extract and ethanol mixture were pipetted into the preconditioned purification column. RNA was bound to the column by centrifuging for 2 min at 100 × *g*, immediately followed by a centrifugation at 16,000 × *g *for 30 sec to remove flow through. One hundred μL wash buffer 1 was pipetted into the purification column and centrifuged for 1 minute at 8,000 × *g*. Contaminating DNA was removed by DNase treatment. One hundred μL of wash buffer 2 (W2) was pipetted into the purification column and centrifuged for 1 min at 8,000 × *g*. Another 100 μL W2 was pipetted into the purification column and centrifuged for 2 min at 16,000 × *g*. The purification column was checked for any residual wash buffer by re-centrifuging at 16,000 × *g *for 1 min and transferred to a new 0.5 mL micro centrifuge tube provided in the kit. Eleven μL of elution buffer was pipetted directly onto the membrane of the purification column and finally, the isolated total RNA was preserved in the elution buffer and used for down stream applications.

RNA quality and yield of each sample were determined using Bioanalyzer 2100 and RNA 6000 Pico LabChip assay (Agilent Technologies Inc, Palo Alto, CA) in combination with Quant-iT™ RiboGreen Reagent according to supplied protocols (Invitrogen, Carlsbad, CA). Initial total RNA concentration across all samples were adjusted to the lowly concentrated sample (12 ng) and this amount of total RNA were used for the two round cDNA synthesis and subsequent in vitro-transcription according to the two-cycle eukaryotic target labeling assay (Affymetrix expression analysis technical manual: Eukaryotic sample and array processing http://www.affymetrix.com/support/technical/manual/. MEGA script in vitro transcription kit containing T7-Oligo (dT) primer and other components (MEGAscript^® ^high yield transcription kit, Applied Biosystems, Ambions) and random primers (Invitrogen, Karlsruhe, Germany) were used for the first and second cycle of cDNA synthesis. Fifteen μg of fragmented and biotin-labeled complementary RNA (cRNA) of each group were hybridized with Affymetrix bovine Genome 430 v2.0 GeneChip^® ^arrays for 16 hrs at 45°C. Post-hybridization staining and washing were performed according to manufacturer's protocols using the Fluidics Station 450 instrument.

### Image capturing, quantification and data analysis

Array slides were scanned with a GeneChip™ 3000 laser confocal slide scanner (Affymetrix) and the images were quantified using Gene Chip Operating Software (GCOS, Affymetrix) version 1.2. Probe level data were imported into the R software environment http://www.r-project.org. Data normalization and background correction were performed using guanine-cytosine Robust Multichip Average (gcRMA) function as described in [54]. gcRMA adjusts for background intensities in Affymetrix array data which include optical noise and non-specific binding (NSB). The main function gcRMA converts background adjusted probe intensities to expression measures using the same normalization and summarization methods as RMA (Robust Multiarray Average). It uses probe sequence information to estimate probe affinity to non-specific binding (NSB). The sequence information is summarized in a more complex way than the simple GC content. Instead, the base types (A,T,G or C) at each position (1-25) along the probe determine the affinity of each probe. The parameters of the position specific base contributions to the probe affinity are estimated in an NSB experiment in which only NSB but no gene-specific binding is expected.

The presence or absence of genes were detected using microarray suit 5 (MAS 5, Affymetrix) as described in [42,55]. To minimize false positive signals, genes called absent were avoided and these called present in at least two of the three replicates were used for further analysis. Differential gene expression was analyzed using linear models for microarray (LIMMA) as described in [56]. LIMMA is a package for differential expression analysis of data arising from microarray experiments. The package is designed to analyze complex experiments involving comparisons between many RNA targets simultaneously while remaining reasonably easy to use for simple experiments. The central idea is to fit a linear model to the expression data for each gene. The expression data can be log-ratios, or sometimes log-intensities, from two colour microarrays or log-intensity values from one channel technologies such as Affymetrix. The MIAME guidelines with details of sample preparation, experimental design, array processing and hybridization, measurements and normalisation controls are given in additional file 1.

Differentially expressed genes in experiments 1A and B were classified according to their gene ontology (GO) using GO consortium [57] and lists of genes over expressed in OO + CCs and CCs + OO relative to those expressed in OO - CCs and CCs - OO respectively, were uploaded into Ingenuity Pathways Analysis (IPA), (Ingenuity Systems, http://www.ingenuity.com) to identify relationships between the genes of interest and to uncover common processes and pathways in the positive phenotypes. IPA is a web-based software application that enables the modelling and analysis of biological systems using microarray data.

### Validation of the microarray data using semi quantitative and quantitative real time RT-PCR

Array data was validated using real time quantitative reverse transcription PCR (qRT-PCR). For this, additional pools of biological replicates representing independent oocytes and CCs samples (n = 150) were used for total RNA isolation and cDNA synthesis. In order to reduce the variability in the concentration of the initial RNA populations across samples and to use

equivalent RNA quantities to the ones used for array hybridization, 12 ng of the total RNA of each sample was used for cDNA synthesis using oligo (dt)25 and random primer as described elsewhere [58]. The ABI prism^® ^7000 apparatus (Applied Biosystems) was used to perform the quantitative analysis using SYBR^® ^Green Jumpstart™ Tag Ready Mix™ (Sigma) incorporation for dsDNA specific fluorescent detection dye. Standard curves were fitted for both target genes and internal control (18S rRNA) using serial dilutions of plasmid DNAs containing 10^1^-10^9 ^molecules and run in separate wells. PCR was assembled using 20 μl total reaction volume containing double distilled (dd) water, forward and reverse primers, SYBR green universal master mix (Sigma) and 2 μl template cDNAs using five replicates for each sample. During each reaction, samples from the same cDNA were run in duplicate to control the reproducibility of the results. A universal thermal cycling parameter (initial denaturizing step at 95°C for 3 min, 45 cycles of denaturizing at 95°C for 30 sec and 58°C for 30 sec were used to quantify mRNA expression level. After the end of the last cycle, dissociation curves were fitted by starting the fluorescence acquisition at 60°C and taking measurements every 7 sec interval until the temperature reaches 95°C. Final quantitative analysis was done using relative standard curve method and the expression values of the target transcripts were normalized to that of 18S. The mean normalized data were reported as the amount of a given target gene transcript in the two samples compared and significantly different means were identified using t-test (SPSS Inc. Chicago, IL, USA). Pairs of primers that were used for array validation are shown in Table 1.

**Table 1:** Pairs of primers that were used for validation of array data

Gene name	Accession number	Forward (5' - 3')	Reverse (3' - 5')	Annealing temperature (°C)	Product length (bp)
GDF9	NM_174681	AGCGCCCTCACTGCTTCTATAT	ACACCCTCAGCAGCTTCTTCTC	57	152
MSX1	NM_174798	AAGGTATCCACAGTCCCCAGC	TCTGCCTCTCCTGCAAAGTTC	56	180
IRF6	NM_001076934	GGACTCCAAACGCTTCCAGA	TCCTTGGTGCCATCATACATCA	54	212
SPARC	NM_174464	CGATGATGGTGCTGAGGAAA	TGGTGGCAAAGAAGTGGCA	53	220
HPSE	NM_1744082	ATGGGCATAGAAGTGGTGATGA	TGTTTGGTGTTTGTGCAATGAA	TD 54-50	202
DGAT2	NM_205793	TGAACCGGGACACCATAGACTA	CACCTCATTCTCCCCAAAGGA	55	205
SOX2	NM_001105463	GCGGCAACCAGAAGAACAG	GCTTCTCCGTCTCGGACAAA	55	170
HAS2	NM_174079	CCAAATGAAATGCCAAAGGAA	CAACGTCAACCAAGCTTCACA	TD 54-50	237
PTX3	NM_001076259	TTTATTCCCCATGCGTTCCA	CTCCACCCACCACAAGCATT	53	205
IGF2BP3	XM_588560	GACGCGAAAGTGAGGATGGT	TGCACTTGACAAATTCTGGAGC	54	215
ADAMTS1	NM_001101080	TCGTCATACAGCTCCCCTCC	ATTGACACACCATTTCCCCTCT	TD 57-54	220
PDK4	XM_583960	ATTTTTGCGACAAGAGTTGCCT	GGATTCCTTGTGCCATTGTAGG	TD 56-52	250
CCRK	XM_879150	GGCCCCACTCATGGCTACTT	TCCTGAGGGTGATGCTGGTAA	57	170
FOSB	XM_880646	TTCCTGAATCTCTCCCGCC	TGCTCACAGCCTCACACTCG	56	205
POU5F1	NM_174580	AGAAGGGCAAACGATCAAGC	GGTGACAGACACCGAGGGAA	TD 57-53	205
VEGFA	NM_174216	GGTTTCGGGAACCAGACGT	GGCAATCCAATTCCAAGAGGA	55	233
18S	NR_003286	GTGCCCTTCCGTCAATTCCT	ACGAAAGTCGGAGGTTCGAA	55	184
GPT	XM_585516	CCGATGAGGTGTACCAAGACAA	CCATATTCACCACCTCCACGT	55	185
DAPL1	NM_001025346	AAATTTCCAGCAGTAGCGCAC	AGCTCTCAGACATTCGAGGCA	55	158
IRF7	NM_001105040	CTCCCCGCACTACACCATCTA	GCCTGTTCCACCTCCATCA	TD 58-55	245
GADD45A	NM_001034247	GACCGAAAGGATGGATAAGGTG	TGGATCAGGGTGAAGTGGATCT	56	200
DPP4	NM_174039	CAACTGGGCTACTTACCTTGCA	TTACGTACCCTCCGTATGACCA	56	223
IFI6	NM_001075588	ACGGTGACAAAGCCTTGAGC	AGGTCACCATGCCCCAGAA	55	158
ADAMTS4	NM_181667	CCATTGTGGAGGATGATGGG	AGGAAGTCAGTGATGAAGCGG	55	210
CASP1	XM_592026	CTCACTCAGAGCATCGGACCT	GTTTACCCATACCATCCCTTGC	57	229
IFIT5	NM_001075698	CACAGTGTATCGGCTGGATGA	GGTTGGGCTGATATCTGGTCC	56	200
DDX39	NM_001034752	GCAGTTCAAGGACTTCCAGC	GCTCTGCCACATTCACTTCA	TD 55-52	228
GTF2A2	NM_001037619	AGAAAGAGGTTCTGCCCGGA	CTTATCGGCTGCATTGAAAGC	55	150
TSSC1	XM_001789233	AGCAGCGACAGCAGAGTCATC	TAGCTCAGGGAGGCGAACAG	57	229
FSHR	NM_174061	GCTGGATCTTTGCTTTTGCAGT	ATGCGCTTGGCTATCTTGGT	54	243
GPT	NM_001083740	CCGATGAGGTGTACCAAGACAA	CCATATTCACCACCTCCACGT	56	185
PLAUR	NM_174423	GGATTCCACAACAACCACACCT	TCGCTTCCAGACATTGATTCAT	TD 56-52	204
CA2	NM_178572	CAAAGCAGTGCTGAAAGATGGA	AAAACACCCACAACAGCCAGTC	55	212

Touch down (TD) = The forward and the reverse primers of a given gene have different annealing temperatures

The appropriateness of 18S as internal control was confirmed by the results of semi-quantitative RT-PCR showing its stability across all samples.

### Immunofluorescence staining

In order to localize the proteins of some differentially expressed transcripts, ovarian sections were washed three times in PBS and fixed in 4% (w/v) Paraformaldehyde overnight at 4°C. The fixed specimens were permeabilized during 2.5 hr incubation in 0.5% (v/v) Triton-X100 (Sigma) in PBS. To inhibit NSB of the antibodies, samples were subsequently blocked in 3% (w/v) bovine serum albumin (BSA) in PBS for 1 hr. The sections were then incubated for 1 hr at 39°C and mounted onto glass slides with gelvatol. The primary antibody for IRF6 (rabbit anti-human polyclonal antibody, Santa Cruz Biotechnologies Inc., Germany) was used at 1:100 in PBS and for MSX1 (rabbit anti-human polyclonal antibody, Lifespan Biosciences, USA) at 1:50 in blocking solution. The samples were incubated for 15 and 1 hr with primary and secondary antibodies (FITC conjugated goat anti-rabbit secondary antibody (Lifespan Biosciences, USA), respectively using 1:100 ratio in both cases. Negative controls were processed in the same manner by omitting the use of primary antibody. In order to visualize the nucleus, the sections were finally incubated in 0.1 mg/ml 4'-6-Diamidino-2-phenylindole (DAPI, Sigma) or propidium iodide (Sigma). After the final wash in PBS, the sections were mounted on glass slides and visualized on ApoTome microscope (ApoTome MicroImaging, Inc., Carl-Zeiss, Germany).

## Results

### Specific transcription programs are exhibited by bovine oocytes and CCs

**Figure 3:**
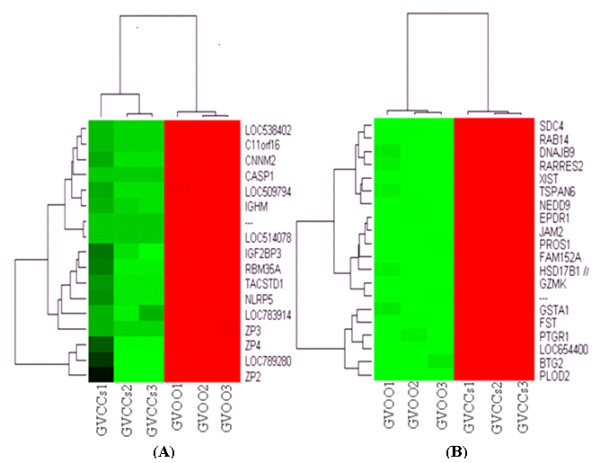
**Hierarchical clustering and heat map of some of the top genes over expressed in GV oocytes (A) and CCs (B) with a fold change of more than 1024**. Abbreviations, GVOO and GVCCs stand for germinal vesicle oocyte and cumulus cells, respectively. Numbers (1, 2, and 3) indicate the three biological replicates used for microarray hybridization.

**Figure 4:**
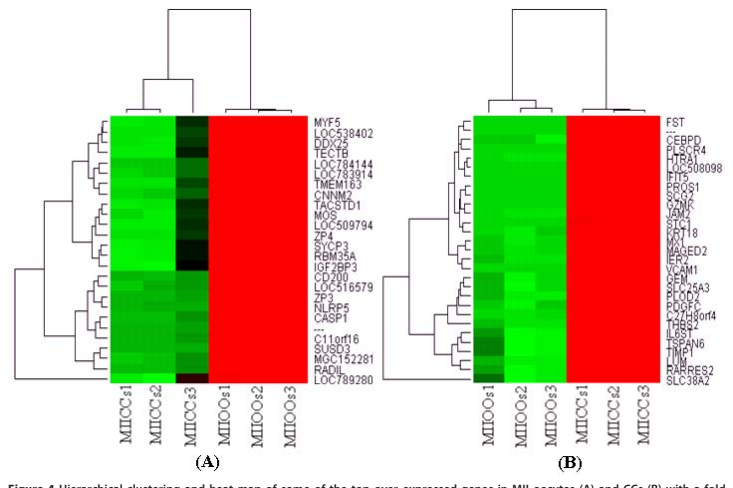
**Hierarchical clustering and heat map of some of the top over expressed genes in MII oocytes (A) and CCs (B) with a fold change of more than 512**. Abbreviations, MIIOO and MIICCs stand for metaphase II oocyte and cumulus cells, respectively. Numbers (1, 2, and 3) indicate the three biological replicates used for microarray hybridization.

In order to get an insight into specific transcription program in bovine oocytes and CCs, we analyzed transcriptome profile of GV (number 1) and MII (number 4 ) oocytes and their companion CCs (number 2 and 3, respectively) using MAS 5 present or absent call as described elsewhere [42,59]. The raw data from all arrays are available online at http://www.ncbi.nlm.nih.gov/geo/ with GEO accession number GSE21005. The analysis showed that of 13162 detected genes, 1516 and 2727 are exclusively expressed in GV oocytes (number 1) and their companion CCs (number 2), respectively while 8919 are expressed in both (Additional files, 2, 3 and 4). Similarly, of 13602 detected genes, 1423 and 3100 are expressed exclusively in MII oocytes (number 4) and their companion CCs (number 3), respectively and 9079 are expressed in both (Additional files, 5, 6 and 7). In addition, expression analysis of these detected genes showed that a total of 8612 transcripts are differentially expressed between GV oocytes (number 1) and CCs (number 2) of which 4304 and 4308 are over expressed in oocytes and CCs, respectively (Additional file 8). Similarly, a total of 8863 transcripts are differentially expressed between MII oocytes (number 4 ) and CCs (number 3) of which 4271 and 4592 were over expressed in MII oocytes and CCs, respectively (Additional file, 9). The heat map and hierarchical clustering of some of the top differentially expressed genes between oocytes and CCs at the two stages are presented in Figures 3A and 3B and 4A and 4B.

**Figure 5:**
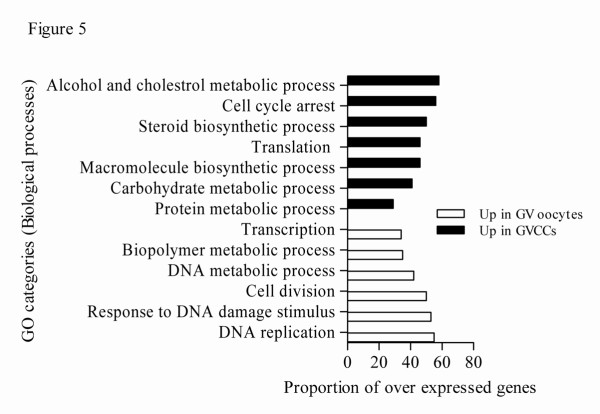
**The top significantly changed GO terms (biological processes) with the proportion of transcripts involved among those over expressed in GV oocytes and CCs**. The proportion of transcripts in a GO term was calculated as the number of genes over expressed in one sample divided by the total number of genes that are involved in that given GO term multiplied by 100 (P < 0.001).

The GO categories (biological and molecular functions) of transcripts over expressed in GV oocytes and CCs relative to each other are shown in Figure 5 and additional file 10, respectively. The most significantly changed molecular and cellular functions associated with genes over expressed in MII CCs relative to their enclosed oocytes and vice versa are also shown in additional file 11.

### Removal of oocyte or CCs at GV alters the gene expression of either cell at MII stage

In order to investigate transcriptome profile changes when the oocyte matures with or without its companion CCs and when CCs mature with or without their enclosed oocytes, we analyzed their corresponding transcriptome profiles using LIMMA as described previously [56]. The analysis showed that a total of 265 genes are differentially expressed between OO + CCs (number 5) and OO-CCs (number 6) of which 217 and 48 are over expressed in OO + CCs and OO - CCs, respectively (Additional file 12).

**Figure 6:**
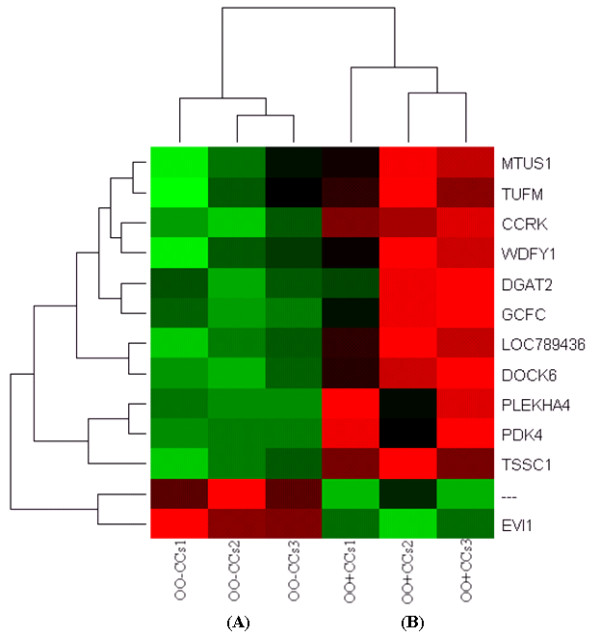
**Hierarchical clustering and heat map of the top differentially expressed genes between oocytes cultured with (red bar) or without (green bar) their companion CCs with a fold change of more than 4**. Abbreviations, OO + CCs and OO - CCs stand for oocytes cultured with or without their companion CCs, respectively and numbers (1, 2 and 3) indicate the three biological replicates that were used for microarray hybridization.

**Figure 7:**
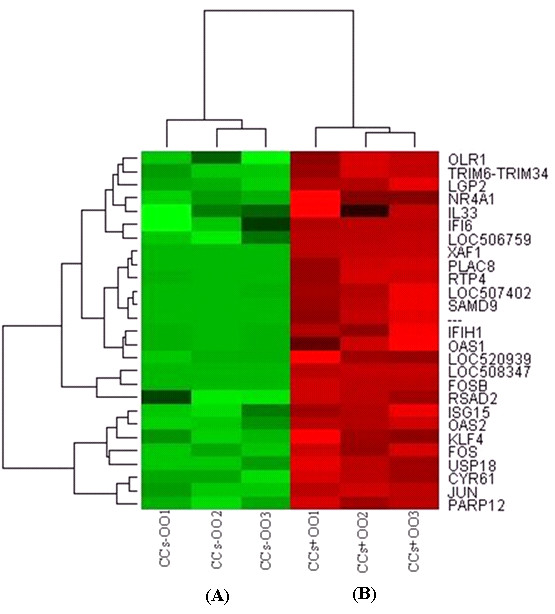
**Hierarchical clustering and heat map of the top differentially expressed genes between CCs cultured with (red bar) or without (green bar) their enclosed oocytes with a fold change of more than 16**. Abbreviations, CCs + OO and CCs - OO stand for cumulus cells cultured with or without ooplasm, respectively. Numbers (1, 2 and 3) indicate the three biological replicates that were used for microarray hybridization.

Similarly, 566 genes are differentially expressed between CCs that were cultured with (CCs + OO) (number 7) or without their enclosed oocytes (CCs - OO) (number 8) of which 320 and 246 are over expressed in CCs + OO and CCs - OO, respectively (Additional file 13). Hierarchical clustering and heat map of the top differentially expressed genes between OO + CCs and OO - CCs and between CCs + OO and CCs - OO, with a fold change of > 4 and 16, are presented in Figures 6 and 7, respectively.

**Figure 8:**
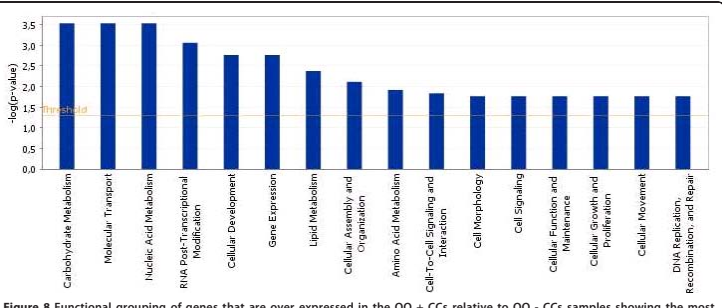
**Functional grouping of genes that are over expressed in the OO + CCs relative to OO - CCs samples showing the most significant functional groups, with P values, 0.05**. The bars represent the P-value in logarithmic scale for each functional group.

**Figure 9:**
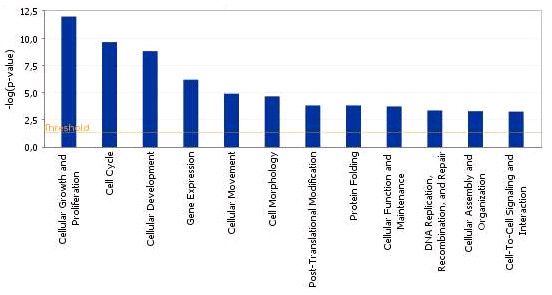
**Functional grouping of genes that are over expressed in the CCs + OO relative to CCs - OO samples showing the most significant functional groups, with P values, 0.05**. The bars represent the P-value in logarithmic scale for each functional group.

**Figure 10:**
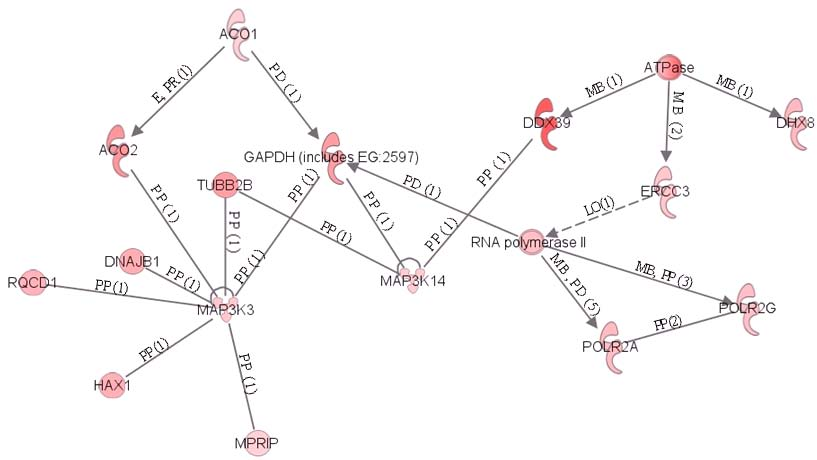
**An example of a gene network, showing the relationships between molecules identified by the microarray and over expressed in oocytes cultured with their companion CCs**. The type of the association between two molecules is shown as a letter on the line that connects them. The number in parenthesis next to the letter represents the number of bibliographic references currently available in the Ingenuity Pathways Knowledge Base that support each one of the relationships. Direct or indirect relationships between molecules are indicated by solid or dashed lines connecting them, respectively. P = phosphorylation, A = gene activation, E = increase in expression, PP = protein-protein interaction, PD = protein-DNA binding, MB = membership in complex, LO = localization, L = proteolysis, RB = regulation of binding

**Figure 11:**
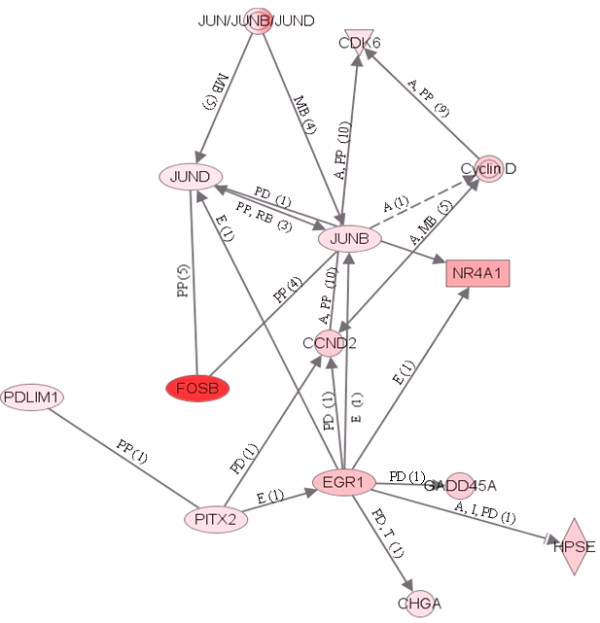
**An example of a gene network, showing the relationships between molecules identified by the microarray and over expressed in CCs cultured with the enclosed oocytes relative to those cultured without**. The type of the association between two molecules is shown as a letter on the line that connects them. The number in parenthesis next to the letter represents the number of bibliographic references currently available in the Ingenuity Pathways Knowledge Base that support each one of the relationships. Direct or indirect relationships between molecules are indicated by solid or dashed connecting lines, respectively. P = phosphorylation, A = gene activation, E = increase in expression, PP = protein-protein interaction, PD = protein-DNA binding, MB = membership in complex, LO = localization, L = proteolysis, RB = regulation of binding

We found that 36 and 375 of these genes over expressed in OO + CCs and CCs + OO, respectively, could be assigned to a specific functional group based on the information in the IPA Knowledge Base. Only 4 of the mapped genes over expressed in OO + CCs group, representing about 1.5% of the total, are classified under the functional group "Carbohydrate metabolism," which contains genes involved in energy conversion and modulation. Other functional groups, including molecular transport, nucleic acid metabolism, small molecule biochemistry and RNA post transcriptional modification are also observed. Similarly, 90 of the genes over expressed in CCs + OO, representing 34% of the total are classified under cellular growth and proliferation. A graphical representation of this functional classification of the genes over expressed in OO + CCs and CCs + OO are shown in Figures 8 and 9, in which 16 and 12 functional groups with higher *P*-values are noted. Some of these groups shared several common genes. In addition, 28 and 23 of the genes over expressed in OO + CCs and CCs + OO relative to OO - CCs and CCs - OO respectively, are assigned to 5 and 8 different canonical pathways (Additional files 14 and 15). Finally, these genes from the two groups were mapped on 5 top networks each network containing genes from the input data that shared known direct or indirect relationships. Examples of networks created from our data are shown in Figures 10 and 11, where the relationships between molecules that were over expressed in OO + CCs and CCs + OO are represented by the arrows that connect them. Figure 10 shows a complex network that plays an important role in gene expression, small molecule biochemistry and carbohydrate metabolism while Figure 11 shows a network that plays a role in cellular development.

### Global transcriptome changes in the CCs and associated functional changes during COCs in vitro maturation

**Figure 12:**
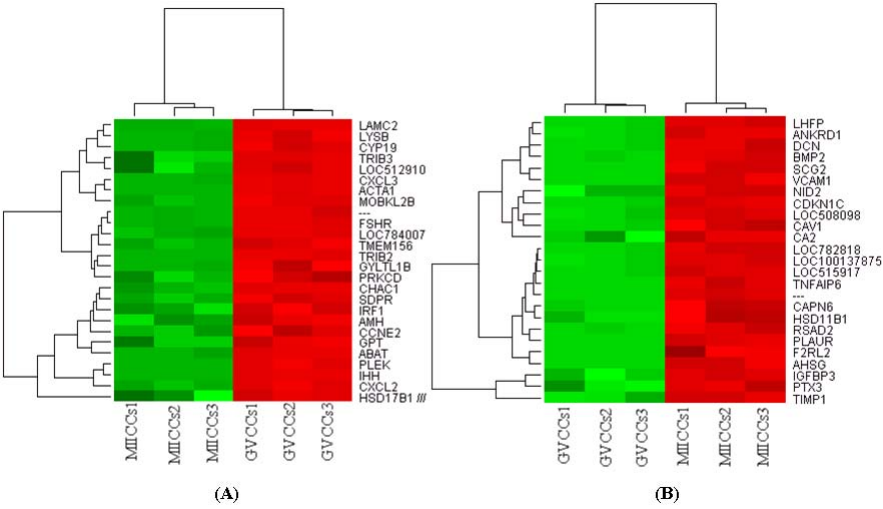
**Hierarchical clustering and heat map of some of the top differentially expressed genes between GVCCs (A) and MIICCs (B) with a fold change of more than 256**. Abbreviations, GVCCs and MIICCs stand for germinal vesicle and metaphase II CCs respectively. Numbers (1, 2 and 3) indicate the three biological replicates that were used for microarray hybridization.

**Figure 13:**
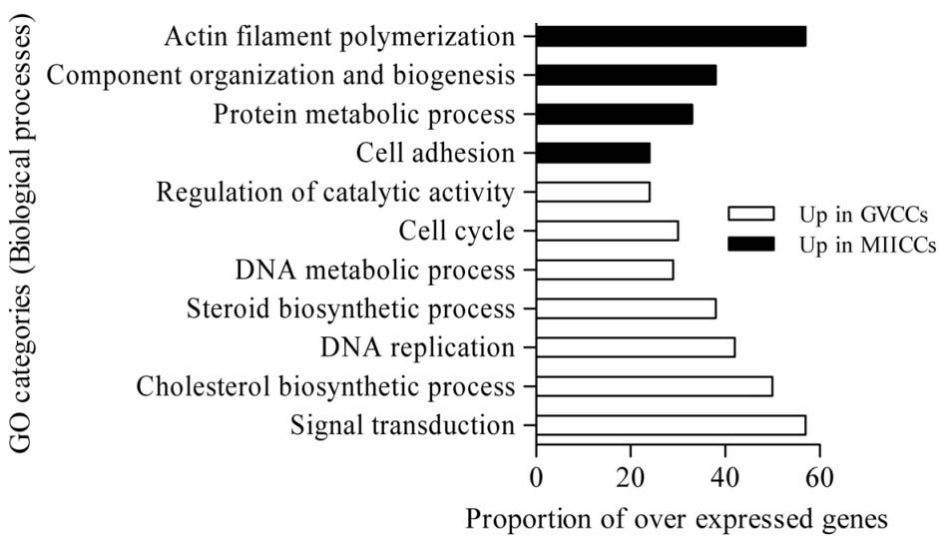
**The top significantly changed GO terms (biological processes) with the proportion of transcripts involved among these over expressed in GV and MIICCs relative to each other**. The proportion of transcripts in a GO term was calculated as the number of genes over expressed in one sample divided by the total number of genes that are involved in that given GO term multiplied by 100 (P < 0.001).

**Figure 14:**
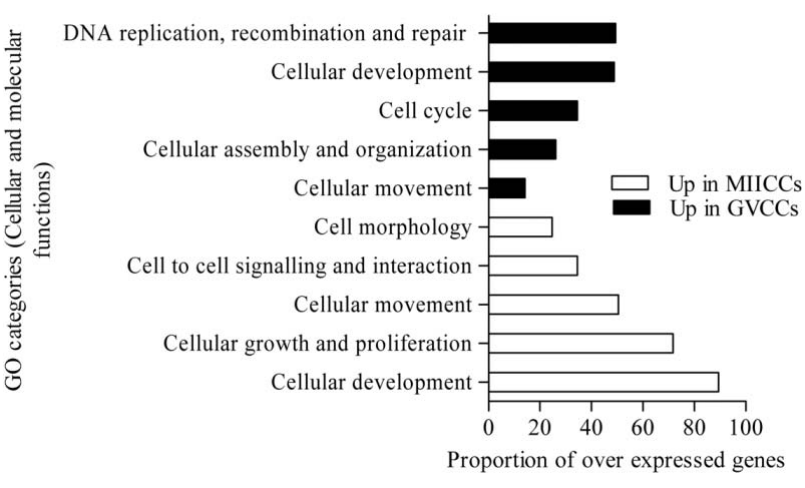
**The top significantly changed GO terms (cellular and molecular functions) with the proportion of transcripts involved among these over expressed in GV and MII CCs relative to each other**. The proportion of transcripts in a GO term was calculated as the number of genes over expressed in one sample divided by the total number of genes that are involved in that given GO term multiplied by 100 (P < 0.001).

In this experiment we analyzed global transcriptome changes during the transition of CCs from GV (number 9) to MII (number 10) stage. The results of MAS 5 present and absent call showed that of 12827 detected genes 4689 and 834 are exclusively expressed in GV and MII CCs, respectively, while 7304 are expressed commonly at both stages (Additional files, 16, 17 and 18). Additionally, expression analysis of these detected genes showed that a total of 4677 genes are differentially expressed between the two samples of which 2397 and 2280 are over expressed in GV and MII stages, respectively (Additional file 19). The heat map and hierarchical clustering of the top differentially expressed genes (fold change > 256), GO categories (biological processes and cellular and molecular functions) between the two CCs stages are shown in Figures 12, 13 and 14, respectively.

### Validation of the microarray data using quantitative and semi quantitative real time RT-PCR

**Figure 15:**
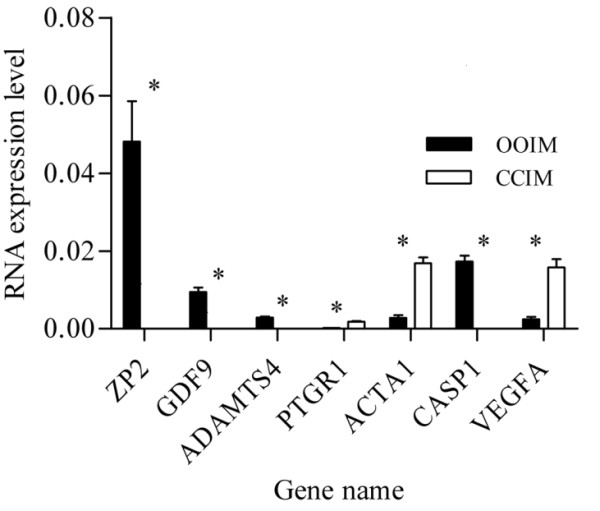
**qRT-PCR validation of the array data showing some selected transcripts that are differentially expressed between GV stage oocytes and CCs**. Two bars representing the same gene and marked with star (*) between them are significantly different (P < 0.05). OOIM = immature (GV) stage oocytes and CCIM = immature (GV) stage CCs.

**Figure 16:**
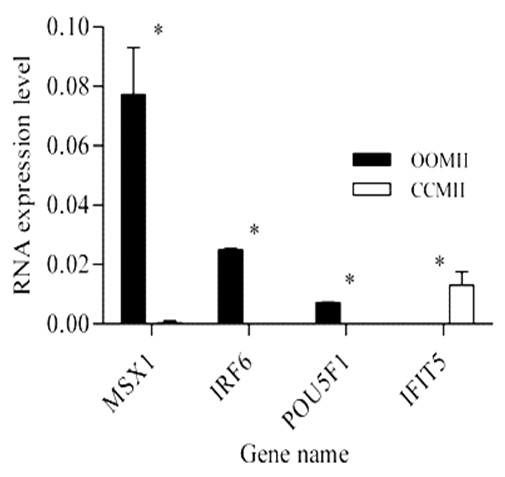
**qRT-PCR validation of the array data showing some selected transcripts that are differentially expressed between MII oocytes and CCs**. Two bars representing the same gene and marked with star (*) between them are significantly different (P < 0.05). OOMII = metaphase II oocytes and CCMII = metaphase II CCs.

**Figure 17:**
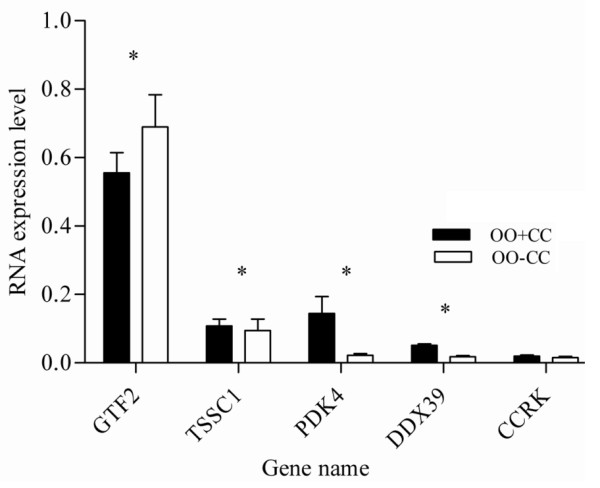
**qRT-PCR validation of the array data showing some selected transcripts that are over expressed in oocytes cultured with their companion CCs compared with those cultured alone**. Two bars representing the same gene and marked with star (*) between them are significantly different (P < 0.05). OO + CC = oocytes cultured with CCs and OO - CC = oocytes cultured without CCs.

**Figure 18:**
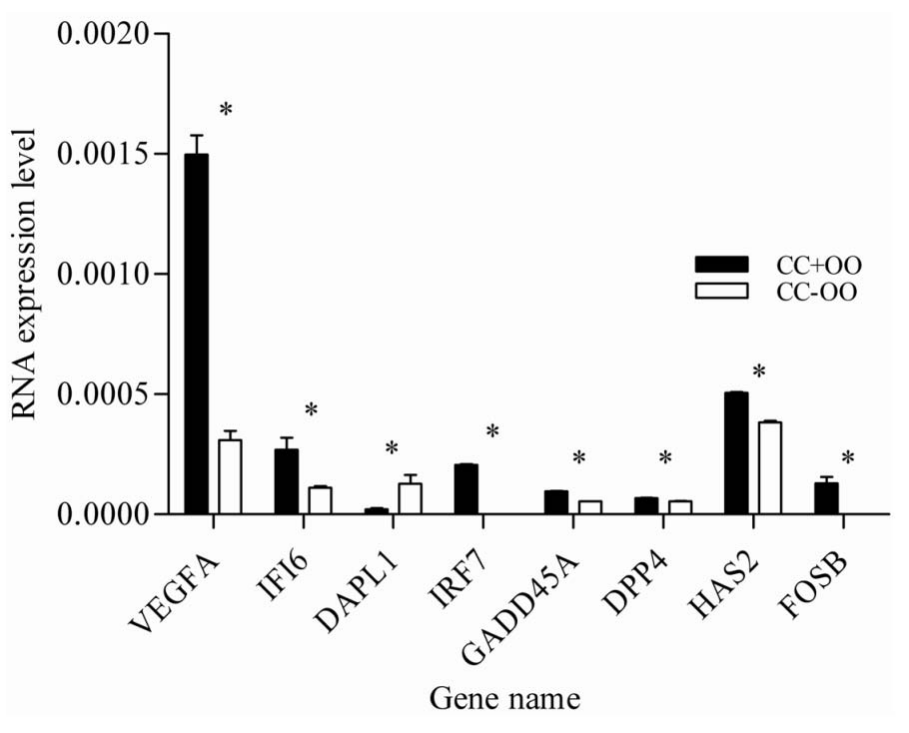
**qRT-PCR validation of the array data showing some selected transcripts that are over expressed in CCs cultured with their enclosed oocytes compared with those cultured alone**. Two bars representing the same gene and marked with star (*) between them are significantly different (P < 0.05). CC + OO = CCs cultured with their enclosed oocyte and CC - OO: CCs cultured without oocyte.

**Figure 19:**
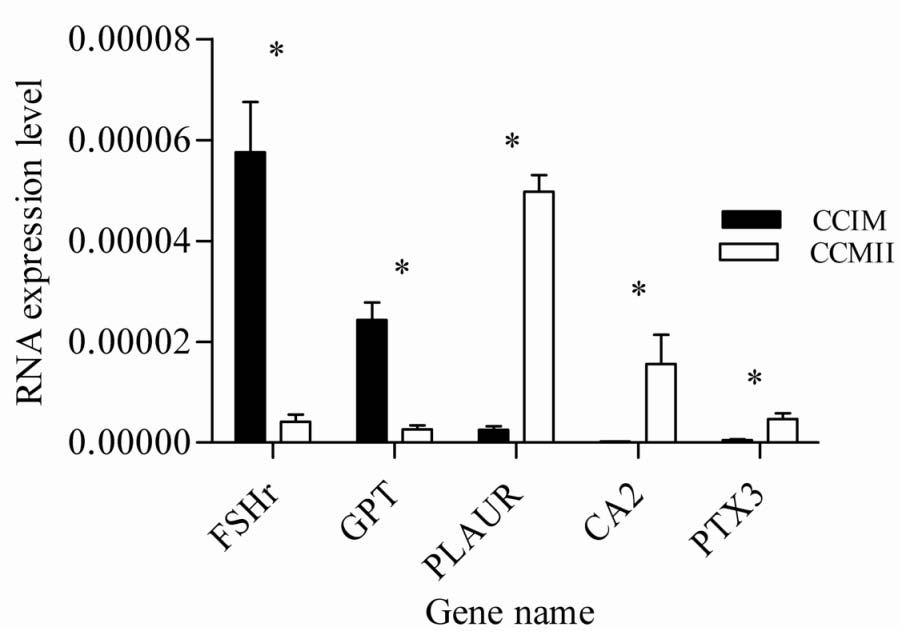
**qRT-PCR validation of the array data showing some selected transcripts that are differentially expressed between GV and MII CCs**. Two bars representing the same gene and marked with star (*) between them are significantly different (P < 0.05). CCIM = immature or GV stage CCs and CCMII = cultured or metaphase II CCs.

**Figure 20:**
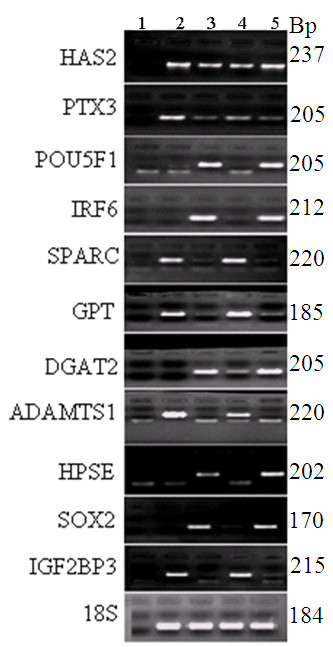
**Validation of the microarray data by semi quantitative RT-PCR. A 2% agarose gel electrophoresis depicting the mRNA expression of genes that are exclusively expressed either in oocytes or CCs**. Number 1 shows a negative control (dd water as a template) and 2, 3, 4 and 5 show the abundance levels of each transcript in MII CCs, MII oocytes, GV CCs and GV oocytes, respectively. 18S was used as a loading control for total RNA.

In order to validate the micro array data, a total of 23 transcripts were quantified using qRT-PCR as described in materials and methods section. The assayed genes are selected based on the criteria of being abundant at significantly higher level or their exclusive abundance in either of the samples considered in the array analysis. Transcripts Semi-quantitative RT-PCR was also adopted to validate some genes that are exclusively expressed either as the oocyte or CC transcripts. With the exception of one transcript, the results of qRT-PCR analysis validated the array data as the expression levels of a given transcript between the two samples compared (designated by *) are significantly different (P < 0.05) (Figures 15, 16, 17, 18, 19). Additionally, semi-quantitative RT-PCR validation of some selected genes also supports the microarray data (Figure 20). The actual microarray expression values of genes validated using real time qRT-PCR analysis are shown as additional files 20, 21, 22, 23 and 24.

**Figure 21:**
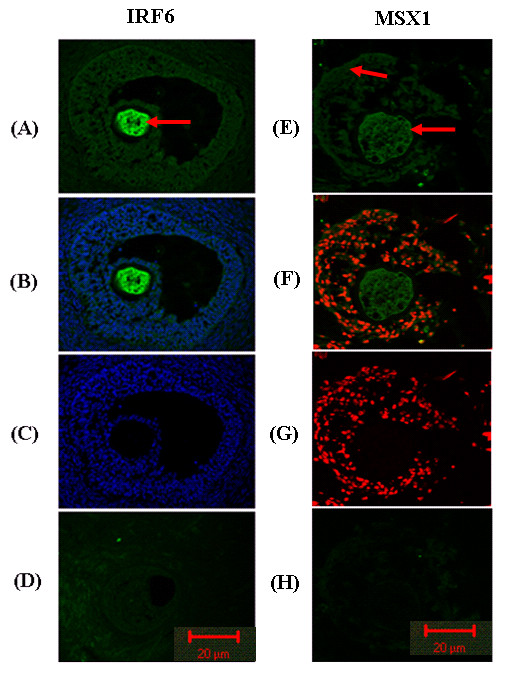
**Immunoflourescence images taken from sections of ovarian follicles showing the protein of IRF6 expressed only in oocytes and that of MSX1 expressed in both oocyte and CCs**. Lanes A and E. = ovarian sections incubated with IRF6 and MSX1 primary antibodies showing the location of IRF6 and MSX1 proteins, respectively. B and F = Protein and nuclear staining showing IRF6 and MSX1 proteins and DNAs together. C = Sections incubated only with DAPI showing the nucleus of the cells D. Negative control staining in which the sections were incubated only with secondary antibodies. The red arrows indicate the location of each protein. Scale bars below each picture represent 20 μm

Immunoflourescence staining for localization of two selected proteins, IRF6 and MSX1, in oocyte or CCs clearly demonstrated that IRF6 is expressed only in oocyte and MSX1 is expressed in both oocyte and CCs showing the accuracy of our hybridization protocol (Figure 21).

## Discussion

We analyzed the transcriptome profiles of bovine oocytes and their companion CCs and investigated transcriptome profile changes when either cell type matures with or without the other using Affymetrix GeneChip Bovine Genome Array. Here, we identified for the first time, transcripts that are exclusively expressed in bovine oocytes or CCs at GV and MII stages. Identifying specific transcription programs either in oocyte or CCs has a paramount importance in RNAi based gene function study. Knowledge of genes exclusively expressed either in oocyte or CCs would enable researchers to select the appropriate design for functional analysis of genes and route of introducing RNAi agents into COC. For instance, if a given gene is expressed only in CCs, transfection is the best method of introducing anti-sense oligomers into the complex. Identification of such genes would help also in understanding functional biological processes and pathways specific to either oocyte or CCs.

Additionally, we assessed the effect of removing the bi-directional communication axis on the gene expression profile of either cell during in vitro maturation and transcriptome profile changes associated with the transition of CCs from GV to MII stage at global scale. Identification of genes that are significantly affected when either the oocyte or CCs mature with or without one or the other would be vital to understand the most important cellular and molecular functions that are associated with the acquisition of developmental competence.

### Specific expression program is exhibited by bovine oocyte and CCs

In addition to the previously identified ones [42,59], this study identified several oocyte or CCs specific transcripts that may play important biological roles in the bi-directional communication of the two cell types during in vitro maturation and for the acquisition of developmental competence at latter stages. Hierarchical clustering of differentially expressed genes demonstrated that the expression profile of oocyte is markedly different from that of its companion CCs with the latter having more number of transcripts than the former.

Transcripts that are over expressed in oocytes are involved in processes leading to meiotic maturation. We found considerably higher (more than 1024 fold change) and exclusive expression of *GDF9, BMP15, MOS*, Zona Ppellucida Proteins (*ZP2, 3, 4*), *NLRP5, RBM35A*, *TACSTD1, GAS7 *and others in oocyte compared with CCs. Since the roles of *GDF9 *and *BMP15 *in oocyte growth and maturation have been widely addressed [2,60,61] we are not going to discuss them here. The *c-mos *proto-oncogene product *MOS *is believed to be an active component of the cytostatic factor that stabilizes and sustains the activity of maturation-promoting factor (MPF). Notable interspecies differences exist among different vertebrates regarding the physiological effects of *MOS *on oocyte maturation. Its higher expression in oocytes both at GV and MII stages in the current study supports previous claims that MOS is required both for the activation of MPF during meiosis I and II and for the meiotic arrest at meiotic MII [62,63].

Trans-membrane proteins are involved in oocyte-granulosa cell regulatory loop and Rho proteins play a role in GTP-bound active state and can interact with a number of effectors to transduce signals leading to diverse biological responses including actin cytoskeletal rearrangements, regulation of gene transcriptions, cell cycle regulation, control of apoptosis and membrane trafficking [64,65]. Phosphorylation and dephosphorylation of proteins are also crucial and control nearly every cellular activities, including metabolism, transcription and translation, cell-cycle progression, cytoskeletal rearrangement, protein-protein interactions, protein stability, cell movement, and apoptosis. These processes in turn depend on the highly regulated and opposing actions of protein kinases (PKs) and phosphatases (PPs) where the balance between the two plays an important role in the control of oocyte meiotic resumption [66]. Consistent with this notion, we found oocyte specific expression of many of the members of trans-membrane proteins (*TMEM30B, TMEM163, TMEM32, TMEM120B *and *TMEM52*) and Rho GTPase activating proteins (*ARHGAP10, 17, 18, 22, 24, 26, 27, 28*), various members of the mitogene activated protein kinases (*MAP4K2, MAPK10, MAPK8IP2*), and phosphatases (*PPP1R1B, PPP2R2B, PPP3R1, PPP1R3D*) that may evidence the roles of these genes in meiotic maturation.

Like wise, some of the transcripts that were highly expressed in CCs relative to oocyte include *IFIT5, BMP2, FSHr, GSTA1, FST, PTGR1*, hormonal receptors and hormones such as *INHA, INHBA, PGR *and *PGRMC2*. The oocyte and CC genes expression study has revealed that the receptor of *BMP2*, also a receptor for *GDF9*, is expressed only in CCs [42]. Similar studies have shown the expression of *BMP2 *receptor in bovine antral follicles and its potential role in the development and functioning of ovarian follicles [21]. In support of these claims, we also detected this gene only in CCs suggesting its higher activity in CCs than in oocyte. *GSTA1 *is highly expressed in steroidogenically active cells of bovine ovarian follicle and suggested to intervene in folliculogenesis and oocyte maturation [67] and steroid receptor cells are found only in CCs evidencing the involvement of CCs derived *GSTA1 *in oocyte maturation. On the other hand, higher expression of *FST *and *INHBA *has been reported in cumulus oophorus that were obtained from in vivo produced COCs compared to these produced in vitro [68].

In order to validate the microarray data, specifically for those transcripts exclusively expressed in oocyte or CCs, we analyzed the expression of some selected genes using semi-quantitative PCR. A 2% agarose gel pictures showing transcripts that are specific to oocyte or CCs are shown in Figure 20. Previous studies have suggested that higher expression of *HAS2, PTX3, TNFAIP6, PTGS2, CD44, INHBA *and *BTC *in CCs can be used as molecular bio-markers to select quality embryo in women [40] and cow [48]. Higher expression of *PTX3, PTGS2, ADAMTS1, INHA *and *INHBA *was also reported in human CCs [42]. But, data that clearly demonstrate whether these transcripts are oocyte or CC specific or expressed in both is not available. Here, we show that none of *HAS2, PTX3, INHA, INHBA *and *CD44 *are CC specific as they are expressed in both samples (supplemental tables S3 and S6, Figure 20).

Additionally, we report for the first time that POU class 5 homeobox 1 (*POU5F1*), interferon regulatory factor 6 (*IRF6*), sex determining region Y box2 (*SOX2*) and insulin like growth factor 2 binding protein 3 (*IGF2BP3*) are expressed only in oocytes while secreted protein, acidic, cysteine-rich (*SPARC*), glutamate pyruvate transaminase (*GPT*), ADAM metallopeptidase with thrombospondin type 1 (*ADAMTS1*) and heparanase (*HPSE*) are expressed only in CCs. Significantly higher expression of *POU5F1 *has been reported in developmentally competent bovine oocytes and its loss of function has resulted in preimplantation lethality in mouse embryos as it is a central regulator of pluripotency [69]. Similarly, *SOX2*, which is expressed only in oocyte, is a developmental pluripotency marker and has been hypothesized as a regulator of *POU5F1 *controlled genes [70] suggesting a possible synergetic effect of the two genes on oocyte maturation.

*IRF6 *has been suggested as a key mediator of cellular proliferation and differentiation in mammary epithelial cells by facilitating entry into the G0 phase of the cell cycle [71]. It regulates cell proliferation and differentiation in different cell types and its higher expression in oocyte sample in the present study at both mRNA and protein levels (Figure 21) may show *IRF6 *as a maternal candidate transcript that play a role in acquiring developmental competence. *ADAMTS1 *is also expressed only in CCs but more abundantly at MII compared to at GV stage (Figure 20). Previously, increased expression of *ADAMTS1 *protein has been reported in mouse GCs in response to preovulatory LH surge [72] where it targets Versican (*VCAN*), one of the proteins that cross link hyaluronic acid (HA) rich CCs matrix and contributes to oocyte maturation, ovulation and/or fertilization [73].

Transcripts specifically expressed in CCs also include SPARC, a multifunctional calcium-binding glycoprotein that modulates extracellular matrix interactions and influences cell-cell adhesion, migration and invasion in vitro and in vivo [74]. Although the role of SPARC in the biology of oocytes is not documented, the fact that it is expressed only CCs supports previous reports where it has been expressed only in the somatic cells of germarium and follicles during oogenesis [74].

### The absence of CCs during in vitro maturation alters the gene expression profile of MII oocyte

Despite the fact that we did not observe significant morphological differences between oocyte matured with or without the surrounding cumulus cells including the polar body extrusion, previous study in bovine [38] has shown that oocytes matured without the surrounding cumulus cells resulted in significantly reduced blastocysts rates compared to those matured in the presence of the surrounding cumulus cells. Therefore, we hypothesized that the absence of cumulus cells during maturation can affect the nuclear and molecular maturation of the resulting oocytes. Maternal gene expression is an important biological process in oocyte maturation. If the oocyte is to complete normal maturation processes, the underlying transcriptional mechanism must be robust. Interestingly, some of the genes that are under expressed due to removal of CCs before in vitro maturation have vital roles in gene expression. The most important one is RNA polymerase II, an enzyme that plays a significant role in gene transcription. Reduced expression of this gene due to removal of CCs before in vitro maturation means the expression of other genes is greatly affected and hence the developmental competence of such oocytes is compromised.

In vitro studies have shown that follicle stimulating hormone (FSH) dependent cyclic adenosine monophosphate (cAMP), the activator of MAPK signaling, is produced by CCs and diffuses to the oocyte via the gap junction [75,76]. The activated MAPK in turn activates the components of MPF to initiate meiotic resumption [77] and simulate mos mRNA cytoplasmic polyadenylation during *Xenopus *[78,79] and mouse [80] oocyte maturation. Low expression of molecules that play roles in biochemistry of oocyte maturation (*MAP3K2, MAP3K3 *and *MAP4K14*) in OO-CCs samples may imply defects in the maturation process due to removal of CCs before maturation.

The capacity of the oocyte to metabolize glucose is positively correlated with its developmental potential and this depends on the presence of companion somatic cells [81,82]. Glucose is a pivotal metabolite for COCs and is metabolized via various pathways. During oocyte maturation, a large proportion of total glucose is metabolized in the CCs via the glycolytic pathway to provide substrates such as pyruvate for energy production [83]. Consistent with this, some genes that are involved in carbohydrate metabolism are under expressed due to removal of CCs indicating defective energy metabolism in the groups cultured without CCs and hence poor developmental competence.

In general, removal of companion CCs at GV stage appeared to affect the gene expression of MII oocytes as a number of genes are over expressed in oocytes cultured with CCs relative to those cultured without. As explained above, some of these genes have been implicated to be involved in various biological processes that are pertinent to oocyte meiotic resumption and maturation supporting the notion that the presence of CCs during in vitro maturation is crucial for oocyte developmental competence. However, the majority of these over expressed genes are uncharacterized and/or their functions, particularly with regard to oocyte development and maturation, are poorly understood. Paradoxically, several previously identified and biologically important OSFs (*GDF9*, *BMP6*, *15*, *TGFBs*), zona pellucida proteins (*ZP2, 3, 4*), the components of MPF (*CDK1 *and *Cyclin B1*) and others are missing from the list of genes over expressed in OO + CCs.

From these results, it can be argued that either these over expressed genes have functional redundancy with those missing genes or the expression of the latter is completed prior to CCs removal at GV stage and consequently they are detected as equally as those cultured without their CCs. One plausible explanation inline of the latter argument is the fact that bovine oocytes are transcriptionally active during folliculogenesis and transcriptional activity decreases at later stages of follicular development [84]. Additionally, our present microarray data analysis between GV and MII oocytes (data not shown) reveals that only *GDF9 *and *CDK1 *are slightly over expressed (fold change = 2.46) at MII relative to GV stage. Interestingly, while *BMP15 *and *TGFB2 *are over expressed at GV stage, *ZP2*, *4 *and *Cyclin B1 *are equally expressed between the two stages. Interestingly several genes have been found to be over expressed in MII stage relative to GV stage including *MPV17, ATP6V1D, TMEM127, NUDT14, UQCR, EXOSC6, MAPK10, CSNK1D, DBNL, FILIP1L, YIF1A and ARHGAP27*. Considering the transcriptional activity of bovine oocytes, the over-expression of transcripts at MII stage compared to the GV stage need further investigation in terms of transcriptional regulation of genes during oocyte maturation.

### The absence of oocyte during in vitro maturation alters the gene expression profile of CCs

Notable interspecies differences exist whether OSFs are mandatory for FSH induced CCs expansion in vitro. In rat, the presence of OSFs are crucial for in vitro CCs expansion as oocytectomized complexes (CCs - OO) failed to expanded their cumulus oophorus [45]. In cattle and pig, CCs expansion doesn't depend on the presence of these factors as CCs - OO expanded as equally as the intact ones [46,47]. In the present study the results of IPA showed that some of the genes under expressed due to removal of oocyte before in vitro maturation are classified into cellular growth and proliferation (*VEGFA*, *GADD45A*, *FOS*, *EGR1*, *HAS2*), cell cycle (*CCND2*, *CDCA8*, *CDK6*), and gene expression (*FOSB*, *TGFB2*, *ATF3*) functional groups (Figure 9). These genes are also mapped into a complex gene network that includes genes involved in cellular development such as *Cyclin D*, *HPSE*, *JUNB *and others (Figure 9). Some of these genes are not well characterized and their direct role in the biology of CC is poorly understood. However, results of previous studies in different species have shown that some of these genes are involved in cumulus expansion and oocyte maturation. For instance, genes from FOS family have been implicated as regulators of cell proliferation, differentiation and transformation and IGFPB proteins stimulate the growth promoting effects of *IGF1 *which in turn is important for oocyte growth and maturation and granulosa cells proliferation [85].

Although a number of genes were differentially expressed between CCs that were cultured with or without their enclosed oocytes, it is not easy to conclude that removal of oocyte completely changes the expression of CCs genes at MII stage. For instance, except *HAS2*, the majority of CCs genes previously identified as molecular bio markers for developmental competence including *INHβA, EGFR, BTC, CD44, TNFAIP6, PTX3 *and *PTGS2 *[40,48] were not differentially expressed between the two sample groups. From these results, we hypothesize that either these differentially expressed genes predict oocyte developmental competence better than the previously indentified ones or the transcription of these previously identified genes is completed earlier at GV stage before the ooplasm is removed and hence they are not differentially expression at MII stage. However, the assertion of both hypotheses requires further investigation.

### The dynamics of CCs transcriptome during the transition of COCs from GV to MII stages is associated with functional changes

Massive transcript degradation during in vitro maturation of bovine [86], human [42,59] and mouse [59] oocytes has been reported. Similarly, considerable shift of transcripts and associated functional changes were observed in CCs during the transition of COCs from GV to MII stage. For instance, while transcripts that are involved in cell cycle, DNA replication, metabolic process, steroid and cholesterol biosynthesis, signal transduction and regulation of catalytic activity are over expressed in CCs at GV stage, these involved in cell adhesion, protein metabolic process, regulation of cellular component organization and biogenesis and actin filament polymerization are over expressed in CCs at MII stage (Figure 13). The most interesting finding of this experiment is not only the change in the number of transcripts but also the identity of transcripts that are involved in a given GO term at the two developmental stages.

Cell cycle and DNA replication are the two successive events which play significant roles in meiotic process resulting in the formation of four haploid cells. The functioning of a cell depends upon its ability to extract and use chemical energy stored in organic molecules via metabolic pathways. On the other hand, MAPK activation in CCs rather than in oocytes exerts essential functions during mammalian oocyte meiotic resumption [66,87] and steroids such as progesterone have been suggested to induce cAMP dependent MAPK signaling cascade leading to meiotic resumption [88]. Hence, over expression of transcripts that are involved in cell cycle, metabolic and steroid biosynthetic pathways in CCs at GV stage suggest that these pathways are more active in GV than in MII stage.

As the oocyte lacks gonadotropin receptors, it has been hypothesized that FSH exerts its effect via a positive meiosis factor (EGF) synthesized by CCs indirectly via signal transduction pathway that involves cAMP dependent MAPK to induce meiotic resumption [89]. From these findings, we propose that over expression of transcripts that are involved in signal transduction pathway at GV stage CCs relative to MII is an indication that this pathway is more active at the former than the latter.

Focal adhesions are large macromolecular assemblies through which both mechanical force and regulatory signals are transmitted [90]. They serve not only to anchor the cell, but also to carry signals, which inform the cell about the condition of the ECM and thus affect their behaviour [91]. In view of their increased expression at MII relative to their expression at GV stage, we propose that these genes are involved in one of the regulatory network that connects the oocyte to its companion CCs during in vitro maturation.

Actin filaments localize to specific regions within mammalian oocytes and their modelling including polymerization are important for oocyte maturation, fertilization and embryo development [76,92,93]. Interestingly, we found higher expression of transcripts that are involved in actin filament polymerization at MII than in GV CCs. Consistent with the notion that in vitro meiotic resumption in bovine oocytes is triggered by FSH [76,93]; we observed higher expression of FSHR mRNA in GV CCs than in MII stage. Prior to in vitro meiotic resumption, FSH is received by CGCs via FSHR and this activates the release of cAMP and MAPK signaling pathways to initiate meiotic resumption [88].

CCs expansion is a key biological event for successful oocyte maturation, ovulation and fertility [40,48,94,95]. *HAS2 *is an important enzyme for the biosynthesis of HA to form stable matrix during CCs expansion [96]. The binding proteins of HA (*TNFAIP6 *and *PTX3*) and its receptor protein (*CD44*) plays significant roles in attaining full CC expansion. *CD44 *is a widely expressed cell adhesion molecule that binds the extracellular matrix component, HA in a tightly regulated manner [97]. The interaction between HA and *CD44 *is the key molecular mechanism for the activation of signalling cascades that contribute to cell adhesion, proliferation, migration and differentiation [98,99]. This interaction is also important for MAPK signalling pathway in oocyte and may promote meiotic resumption [100]. Oocytes can't attain cytoplasmic maturation when cultured in the absence of their companion CCs as they can't store sufficient mRNAs, proteins and transcription factors that are important for maturation process due to removal of the b-idirectional communication axis. The interaction between *HA*-*CD44 *is critical for modification of this communication axis during CCs expansion [101] and relatively higher expression of these molecules in cultured CCs is consistent with the notion that *HA*-*CD44 *interactive effect is vital for oocyte maturation [100].

The majority of genes differentially expressed between different oocyte and CCs samples are poorly characterized and their roles in the biology of bovine oocyte are not known. Moreover, due to the dynamic nature of gene expression in different species, tissues and follicular stages, what have been reported so far in other organisms may not necessarily hold true for bovine oocytes and CCs. Therefore, we recommend detailed gene by gene study to unveil specific roles of these genes in the biology of bovine COCs.

## Conclusion

In conclusion, this study has generated large scale gene expression data from different oocyte and CCs samples that would provide insights into gene functions and interactions within and across different pathways that are involved in the maturation of bovine oocytes. Moreover, the presence or absence of oocyte and CC factors during bovine oocyte maturation can have a profound effect on transcript abundance in each cell types, showing the molecular cross-talk between oocytes and their corresponding CCs.

## Authors' contributions

AR developed the project, carried out the experiment, analysed the data, prepared the drafting of the manuscript and corrected it after revision. FR and MH prepared oocyte and CCs samples for molecular analysis. UC, ET and CL were involved in the discussion of the experimental design and during follow-up of the experiment. KS supervised the work and provided suggestion. DT developed the project and reviewed the manuscript. All authors read and approved the final manuscript.

## Supplementary Material

Additional file 1 **The Six MIAME guidelines that were adopted to conduct the study**. Click here for file

Additional file 2 **List of transcripts that are exclusively expressed in GV oocytes relative to GV CCs**. Click here for file

Additional file 3 **List of transcripts that are exclusively expressed in GV CCs relative to GV oocytes**. Click here for file

Additional file 4 **List of transcripts that are expressed in both GV oocytes and CCs**. Click here for file

Additional file 5 **List of transcripts that are expressed only MII CCs**. Click here for file

Additional file 6 **List of transcripts that are expressed in both MII oocytes and CCs**. Click here for file

Additional file 7 **List of transcripts that are differentially expressed between GV oocytes and CCs**. Click here for file

Additional file 8 **List of transcripts that are expressed only in MII oocytes**. Click here for file

Additional file 9 **List of transcripts that are differentially expressed between MII oocytes and CCs**. Click here for file

Additional file 10 **The top significantly changed GO (molecular functions) with the proportion of transcripts that are over expressed in GV oocytes and CCs (P < 0.001)**. Click here for file

Additional file 11 **The top significantly changed GO (molecular functions) with the proportion of transcripts that are over expressed in MII oocytes and CCs (P < 0.001)**. Click here for file

Additional file 12 **List of transcripts that are differentially expressed between oocytes cultured with or without their companion CCs**. Click here for file

Additional file 13 **List of transcripts that are differentially expressed between CCs cultured with or without their oocyte**. Click here for file

Additional file 14 **The seven most prominent canonical pathways involving genes that are over expressed in OO + CCs relative to OO - CCs with P-values, 0.05**. The bars represent the P-value for each pathway. The orange irregular line is a graph of the ratio (genes from the data set/total number of genes involved in the pathway) for the different pathways. Click here for file

Additional file 15 **The five most prominent canonical pathways involving genes that are over expressed in CCs + OO relative to CCs - OO with P-values, 0.05**. The bars represent the P-value for each pathway. The orange irregular line is a graph of the ratio (genes from the data set/total number of genes involved in the pathway) for the different pathways. Click here for file

Additional file 16 **List of transcripts that are exclusively expressed in GV CCs compared to MII CCs**. Click here for file

Additional file 17 **List of transcripts that are exclusively expressed in MII CCs compared with GV CCs**. Click here for file

Additional file 18 **List of transcripts that are commonly expressed between CCs at GV and MII stages**. Click here for file

Additional file 19 **List of transcripts that are differentially expressed between GV and MII CCs**. Click here for file

Additional file 20 **qRT-PCR validation of the microarray data showing the change in the expression levels of transcripts differentially expressed between GV oocytes and CCs**. Transcripts marked by the minus sign indicate those over expressed in GV CCs. Click here for file

Additional file 21 **qRT-PCR validation of the microarray data showing the change in the expression levels of transcripts differentially expressed between MII oocytes and CCs**. Transcripts marked by the minus sign indicate those over expressed in MII CCs. Click here for file

Additional file 22 **qRT-PCR validation of the microarray data showing the change in the expression levels of transcripts differentially expressed between oocytes cultured with their companion CCs relative to those cultured alone**. Transcripts marked by the minus sign indicate those over expressed in oocytes cultured without their companion CCs. Click here for file

Additional file 23 **qRT-PCR validation of the microarray data showing the change in the expression levels of transcripts differentially expressed between CCs cultured with their enclosed oocytes relative to those cultured alone**. Transcripts marked by the minus sign indicate those over expressed in CCs cultured without their enclosed oocytes. Click here for file

Additional file 24 **qRT-PCR validation of the microarray data showing the change in the expression levels of transcripts differentially expressed between CCs at germinal vesicle (GV) and metaphase II (MII) stages**. Transcripts marked by the minus sign indicate those over expressed in MII stage relative to GV stage. Click here for file
