# Supplementary material for: Transcriptome dynamics and molecular cross-talk between bovine oocyte and its companion cumulus cells
Source: BMC Genomics. 2011 Jan 24;12:57. doi: 10.1186/1471-2164-12-57 (PMC3045333; doi:10.1186/1471-2164-12-57)
Supplement: Additional file 1 — The Six MIAME guidelines that were adopted to conduct the study. [file 1471-2164-12-57-S1.DOC]

**Experimental design:**

18 independent hybridizations representing three technical replications for germinal vesicle stage bovine oocytes (slide number 1, 2 and 3) and GV CCs (slide number 4, 5 and 6) metaphase II oocytes (slide number 7, 8 and 9), and MII CCs (slide number 10, 11 and 12), oocytes cultured without their companion CCs (slide number 13, 14 and 15), CCs cultured without their enclosed oocytes (slide number 16, 17 and 18) were made.

**Array design:** The GeneChip® Bovine Genome Array that can be used to study gene expression of over 23,000 bovine transcripts (Affymerix).

Bos Taurus (Bovine) probe sets 24,072

Bos Taurus (Bovine) transcripts approximately 23,000

UniGene clusters approximately 19,000

**Unique probe sets to single species:**

Number of arrays in set one

Array format 100

Feature size 11 µm

Oligonucleotide probe length 25-mer

Probe pairs/sequence 11

Hybridization controls: bioB, bioC, bioD, from E. coliand cre from

P1 B. subtilis

Poly-A controls: dap, lys, phe, thr, trp from B. subtilis

Housekeeping/Control genes: actin, GAPDH, eflα, 5.8S rRNA, 12S rRNA,

18S rRNA, cyclophilin B, glutathione S-transferase,

lactophorin, translation initiation factor eIF-4E

Detection sensitivity 1:100, 0001

Critical Specifications

**Samples:** the oocytes and CCs samples that were used in this study were obtained from abattoir derived bovine ovaries. Total RNA was isolated from each samples using Pico pure total RNA isolation kit according to the manufacturer’s instruction. Biotin labelled and fragmented cRNA was hybridised in to Affymetrix Bovine Genome Array.

**Hybridization:** hybridization solution containing hybridization cocktail consisting of 10 µg fragmented and biotin labeled cRNA, control oligonucleotide B2 (3 nM), 20x eukaryotic hybridization controls (bioB, bioC, bioD, cre, Affymetrix, CA, USA), 2X hybridization mix, DMSO and RNAse free water were mixed to a final volume of 200 µl and 130 µl of hybridization cocktail was hybridized to the array. Array slides were washed and stained using the Fluidics Station 450 and 250 instruments integrated with GeneChip® Operating System as recommended in GeneChip® expression wash, stain and scan user manual (P/N 702731, Affymetrix).

**Measurements:** A microarray quantification matrix that contains the complete image analysis (the normalized expression values and the raw data and the cell intensity file) has been uploaded to <http://www.ncbi.nlm.nih.gov/geo/> and can be accessed at <http://www.ncbi.nlm.nih.gov/projects/geo/query/acc.cgi?acc> with GEO accession number = GSM524884.

**Normalisation controls:**

- Different control samples were included in the sample target at different phase of hybridization process including:

1. Poly-A controls (lys, phe, thr and dap) which are used as sensitivity indicators of target preparation and labelling efficiency were prepared and added to the total RNA of each sample following the dilution scheme described in affymetrix manual *Eukaryotic Target Preparation (*701025 Rev. 5).
2. To control the hybridization process for troubleshooting, the 20X Eukaryotic hybridization controls consisting of a mixture of biotinylated and fragmented cRNA of bioB, bioC, bioD, and cre in staggered concentrations were added directly to the hybridization cocktail. Control Oligo B2 was also included in the hybridization cocktail to provide alignment signals for image analysis.

- The identities and location of array elements serving as controls and their type is readily available as GeneChip Bovine Genome Array at <http://www.affymetrix.com/Auth/analysis/downloads/na21/ivt/Bovine.na21.blast.csv.zip>

- Elements serving as controls in bovine genome array include:

1. Spike controls (Poly-A controls): dap, lys, phe, thr, trp from B. subtilis

2. Hybridization controls: bioB, bioC, bioD, from E. coli and cre from P1 B. subtilis

3. Housekeeping/control genes: actin, GAPDH, eflα, 5.8S rRNA, 12S rRNA, 18S rRNA, cyclophilin B, glutathione S-transferase, lactophorin, translation initiation factor eIF-4E

Normalization was done based on probe sequence and with GC-content background correction. Starting with the probe-level data from a set of arrays, the perfect-match (PM) values were background-corrected; quantile normalized and finally summarized as the set of expression measures

- The algorism of GCRMA was used to normalise the expression data and arrayQualityMetrics quality control method was used for generation of Boxplots of expression values, MA plots, quality diagnostics based on probe level models.
