# Supplementary figures and images for: Transcriptome dynamics and molecular cross-talk between bovine oocyte and its companion cumulus cells
Source: BMC Genomics. 2011 Jan 24;12:57. doi: 10.1186/1471-2164-12-57 (PMC3045333; doi:10.1186/1471-2164-12-57)

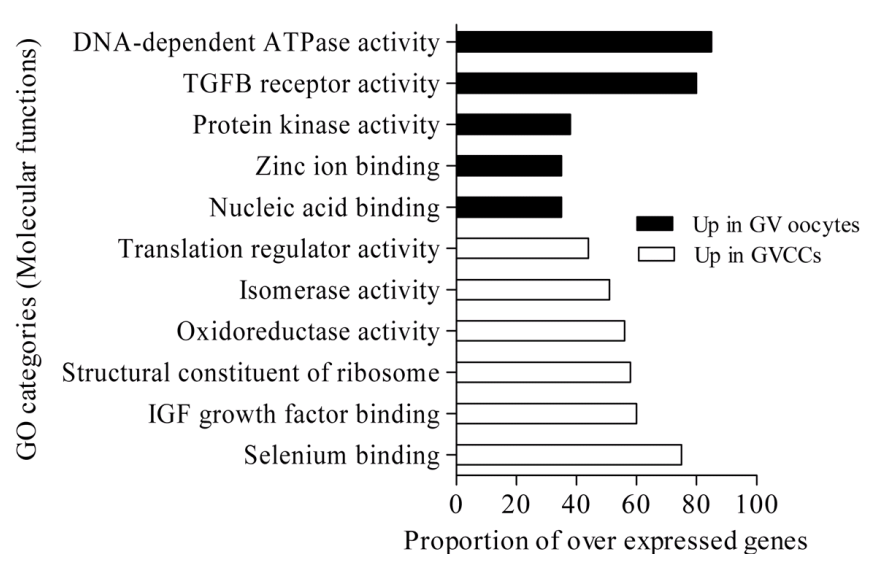

Supplement: Additional file 10 — The top significantly changed GO (molecular functions) with the proportion of transcripts that are over expressed in GV oocytes and CCs (P < 0.001). [file 1471-2164-12-57-S10.TIFF]

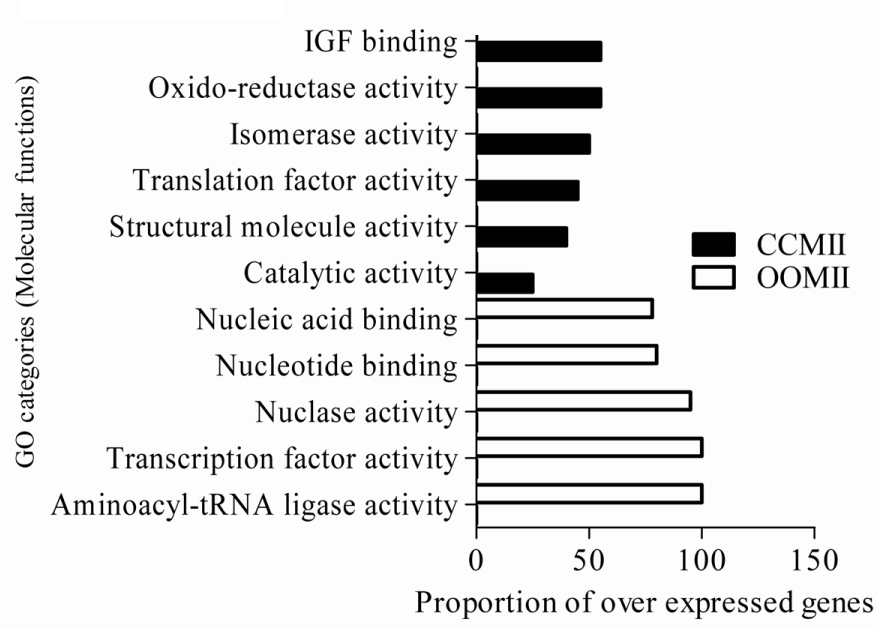

Supplement: Additional file 11 — The top significantly changed GO (molecular functions) with the proportion of transcripts that are over expressed in MII oocytes and CCs (P < 0.001). [file 1471-2164-12-57-S11.TIFF]

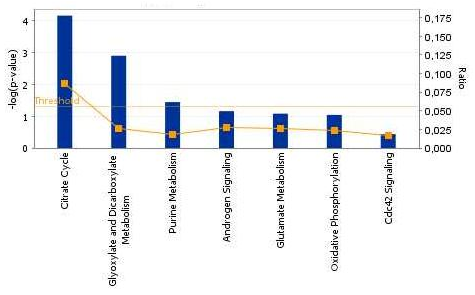

Supplement: Additional file 14 — The seven most prominent canonical pathways involving genes that are over expressed in OO + CCs relative to OO - CCs with P-values, 0.05. The bars represent the P-value for each pathway. The orange irregular line is a graph of the ratio (genes from the data set/total number of genes involved in the pathway) for the different pathways. [file 1471-2164-12-57-S14.TIFF]

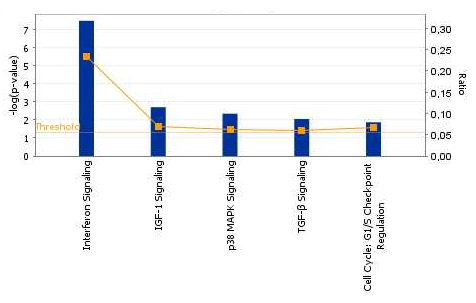

Supplement: Additional file 15 — The five most prominent canonical pathways involving genes that are over expressed in CCs + OO relative to CCs - OO with P-values, 0.05. The bars represent the P-value for each pathway. The orange irregular line is a graph of the ratio (genes from the data set/total number of genes involved in the pathway) for the different pathways. [file 1471-2164-12-57-S15.TIFF]
