# Supplementary material for: Transcriptome dynamics and molecular cross-talk between bovine oocyte and its companion cumulus cells
Source: BMC Genomics. 2011 Jan 24;12:57. doi: 10.1186/1471-2164-12-57 (PMC3045333; doi:10.1186/1471-2164-12-57)
Supplement: Additional file 24 — qRT-PCR validation of the microarray data showing the change in the expression levels of transcripts differentially expressed between CCs at germinal vesicle (GV) and metaphase II (MII) stages. Transcripts marked by the minus sign indicate those over expressed in MII stage relative to GV stage. [file 1471-2164-12-57-S24.DOC]

qRT-PCR validation of the microarray data showing the change in the expression levels of transcripts differentially expressed between CCs at germinal vesicle (GV) and metaphase II (MII) stages. Transcripts marked by the minus sign indicate those over expressed in MII stage relative to GV stage.

| Gene symbol | Microarray | | qRT-PCR | |
| --- | --- | --- | --- | --- |
|  | LogFC | P value | FC | P value |
| FSHr | 6.3 | 1.15E-21 | 13.82006186 | 0.02 |
| GPT | 6.9 | 9.54E-11 | 9.383856105 | 0.03 |
| PLAUR | -8.8 | 5.51E-16 | -19.50230328 | 0.03 |
| CA2 | -8.5 | 2.05E-11 | -81.00343521 | 0.04 |
| PTX3 | -8.8 | 1.36E-11 | -9.677196997 | 0.04 |
